# Functional distinction in oncogenic Ras variant activity in *Caenorhabditis elegans*

**DOI:** 10.1242/dmm.050577

**Published:** 2024-08-14

**Authors:** Haimeng Lyu, Helen M. Chamberlin

**Affiliations:** Department of Molecular Genetics, Ohio State University, 484 West 12th Avenue, Columbus, OH 43210, USA

**Keywords:** *C. elegans* vulva development, Oncogenic Ras variants, Ras/MEK signaling pathway

## Abstract

*Ras* genes are important oncogenes that are frequently mutated in cancer. Human oncogenic variants exhibit functional distinctions in terms of their representation in different cancer types, impact on cellular targets and sensitivity to pharmacological treatments. However, how these distinct variants influence and respond to the cellular networks in which they are embedded is poorly understood. To identify novel participants in the complex interplay between *Ras* genotype and cell interaction networks *in vivo*, we have developed and tested an experimental framework using a simple vulva-development assay in the nematode *C. elegans.* Using this system, we evaluated a set of Ras oncogenic substitution changes at G12, G13 and Q61. We found that these variants fall into distinct groups based on phenotypic differences, sensitivity to gene dosage and inhibition of the downstream kinase MEK and their response to genetic modulators that influence Ras activity in a non-autonomous manner. Together, our results demonstrated that oncogenic *C. elegans* Ras variants exhibit clear distinctions in how they interface with the vulva-development network and showed that extracellular modulators yield variant-restricted effects *in vivo*.

## Teaser

**Summary:** This research uncovers distinct functional classes for oncogenic Ras variants in a *C. elegans* model, including distinctions in their response to genetic modifiers that function in neighboring cells.

## INTRODUCTION

Ras proteins are a class of highly conserved small GTPases that play a critical role in cell cycle regulation and development in animals (reviewed by Pylayeva-Gupta et al., 2011). In humans, the three genes encoding Ras proteins, i.e. *KRAS*, *NRAS*, *HRAS*, are important oncogenes and crucial drivers of many human cancers, especially pancreatic, colorectal and lung (Prior et al., 2012; Waters and Der, 2018). While the clinical importance of oncogenic Ras has been understood for over 40 years, therapeutics that specifically and effectively target Ras have not yet been established as standard-of-care. The overwhelming majority of sporadic oncogenic mutations affecting human *Ras* genes result in amino acid substitutions at just three positions (G12, G13 and Q61), but there is increasing awareness that different oncogenic Ras variants should be evaluated for their distinct properties, rather than considered as a single class of activated *Ras* mutations. This re-framing has yielded a number of important new research directions, including sub-classifying cancers on the basis of sensitivity to established, network-targeting therapeutics (McFall and Stites, 2021) and design of variant- or protein-specific inhibitors (Kim et al., 2023; Ostrem et al., 2013; Wang et al., 2022). While these developments are encouraging, clinical trials for FDA-approved G12C inhibitors report that only a subset of patients exhibit a response, many experience treatment-related adverse events and tumors rapidly evolve resistance (reviewed by Parikh et al., 2022; Rosen et al., 2023). Consequently, it is clear that the cellular networks in which oncogenic Ras proteins are embedded can influence outcomes. Understanding how different variants differentially engage effector and other proteins within a cancer cell, and how they may have distinct roles in the communication between cancer and non-cancer cells, are still open questions. Addressing these questions will be important for targeting Ras-driven cancers in a robust, multi-target manner that can increase drug efficacy and coincidentally reduce toxicity and the capacity of cells to evolve resistance.

**Fig. 1.**
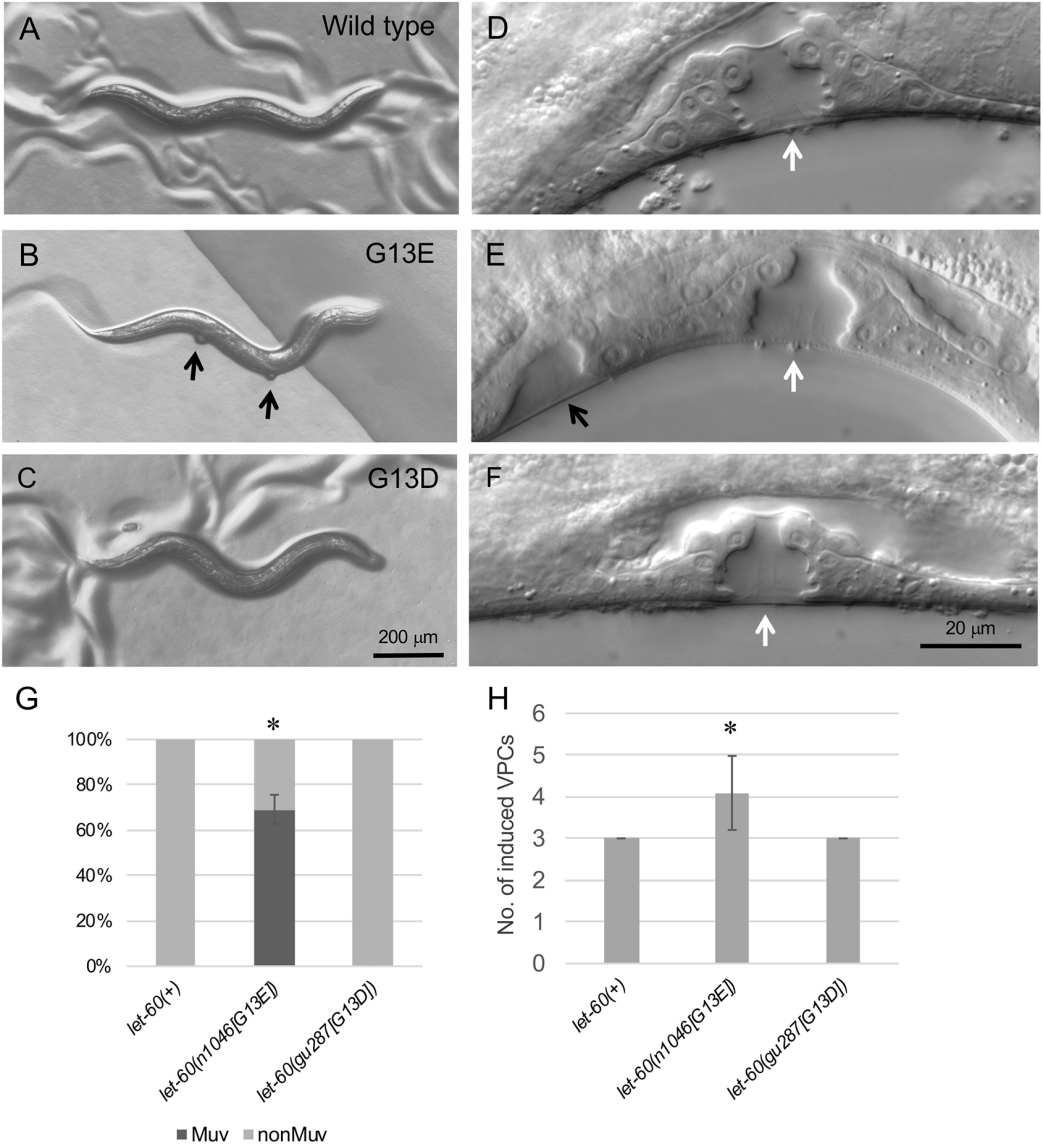
***C. elegans let-60* alleles that code for the oncogenic variant G13D exhibit normal vulva development.** (A-C) Adult wild-type (A) and *let-60(gu287[G13D])* (C) animals exhibit a smooth ventral surface, whereas many *let-60(n1046[G13E])* animals (B) exhibit notable ventral bumps (black arrows) corresponding to the Muv phenotype. Animals are anterior to the right, ventral down. (G) Quantification of experiments described for A-C. Sample sizes in G: *n*=41 for *let-60(+)*; *n*=52 for *let-60(n1046[G13E])*; *n*=45 for *let-60(gu287[G13D])*. (D-F) Vulva structure in animals at larval stage 4 (L4). Wild-type (D) and *let-60(gu287[G13D])* (F) animals exhibit formation of a single vulval opening (white arrow) produced when three of six vulval precursor cells (VPCs) are induced to produce vulval tissue. *let-60(n1046[G13E])* animals (E) frequently exhibit additional invaginations (black arrow) that result when VPCs in addition to the normal three are induced to produce vulval tissue. (H) Quantification of experiments described for D-F. Sample sizes in H: *n*=30 for *let-60(+)*; *n*=55 for *let-60(n1046[G13E])*; *n*=34 for *let-60(gu287[G13D])*. Error bars correspond to standard error of the proportion (G) or standard deviation (H). Asterisks indicate statistical differences compared with wild-type (control) (*P*<0.05); two proportion *Z*-test for G, *t*-test for H.

Experimental animal models will be important in uncovering the complex relationships between Ras variants and other factors that influence Ras activity and disease progression. The nematode *C. elegans* provides a simplified system to study certain aspects of Ras biology in a multicellular context (reviewed by Cerón, 2023; Sundaram, 2013). The *C. elegans* genome includes a single *Ras* ortholog, i.e. *let-60* (Fig. S1), and the gene has been shown to play an important role in development, homeostasis, nervous system function and other processes (Church et al., 1995; Hirotsu et al., 2000; Schutzman et al., 2001; Yochem et al., 1997; reviewed by Sundaram, 2013). *let-60* function has been well-characterized in the development of the hermaphrodite vulva, a specialized epithelial structure that connects the gonad to the outside, permitting egg laying and mating with males (Beitel et al., 1990; Han and Sternberg, 1990). This structure is formed during larval development, where an EGF signal, LIN-3, from the anchor cell in the somatic gonad coordinates development and fate patterning of the six epithelial vulval precursor cells (VPCs) (reviewed by Sternberg, 2005). Normal vulval development occurs when three of six VPCs are induced to divide and produce vulval cell types, utilizing a canonical Ras/Raf/MEK/MPK signaling pathway. Importantly, classic genetic screens have identified gain-of-function alleles of *let-60* that cause increased activity of Ras, resulting in division of up to six of the VPCs (rather than the wild type three), producing a multivulva (Muv) phenotype that causes animals to have visible bumps or protrusions on their ventral sides (Fig. 1B). These genetic mutants have provided the background for genetic dissection of the functional relationship between activated Ras and important effector proteins and downstream targets (Han et al., 1993; Lackner et al., 1994; Sieburth et al., 1998; Singh and Han, 1995; Wu and Han, 1994), for the screening of molecular inhibitors (Reiner et al., 2008; van der Hoeven et al., 2020) and discovery of new genes that mediate or modulate Ras activity (Bruinsma et al., 2002; Corchado-Sonera et al., 2022; Goldstein et al., 2006; Rocheleau et al., 2005; Sundaram and Han, 1995). While studies that used existing gain-of-function alleles have been crucial for understanding the signaling-network response to general Ras dysregulation, they were of limited use in a framework aiming to uncover variant-specific differences. Many *C. elegans* studies utilize gain-of-function alleles that cause a G13E substitution (Beitel et al., 1990), i.e. a variant not observed in human tumor samples (Fig. S1B; GCD database data release 38.0) (Grossman et al., 2016). Thus, it is not clear what the consequences of substitutions that mimic human tumor variants have in *C. elegans* and whether this powerful model system can be extended to variant-specific, rather than simply gene-specific, experimental discovery and chemical compound screening.

Here, we utilized CRISPR-mediated genome editing and controlled, single copy transposon insertion methods to evaluate the function of a set of oncogenic Ras variants in the *C. elegans* vulval development system. We show that, in contrast to the available *let-60(G13E)* mutants, a *let-60(G13D)* substitution did not grossly disrupt vulval development. Using insertions at a Mos1-mediated single-copy insertion (MosSCI) transposon landing site (Frøkjær-Jensen et al., 2014), we assessed the impact on *let-60* gene function of several oncogenic substitution changes at G12, G13 and Q61. Importantly, we found that these substitutions have different effects, including whether they cause a Muv phenotype or whether animals are sensitive to gene dosage. Finally, we showed that the variants exhibit differential response to two non-autonomous modifiers (*hpo-18* and *szy-5*) identified in a genetic screen using *let-60(n1046[G13E])* (Corchado-Sonera et al., 2022). Together these results establish a systematic approach to assay oncogenic Ras variants and uncover functional distinctions among them using *C. elegans* as a model.

## RESULTS

### Isolation and characterization of *let-60(G13D)* mutants in *C. elegans*

With respect to sequence, the human variant most similar to the available *C. elegans let-60(n1046[G13E])* is the smaller, but similarly charged G13D, which is a common substitution at G13 in cancer samples (Fig. S1) (McFall and Stites, 2021). One reason for the difference is suggested by the genomic sequence, as the predicted codon for amino acid 13 differs between *C. elegans* and human – i.e. GGA for *C. elegans* versus GGU or GGC for each human gene – with the G13D substitution requiring a two nucleotide change in *C. elegans*. Consequently, we first asked whether oncogenic variants cause a gain-of-function phenotype in *C. elegans* by directly engineering this substitution into the *let-60* locus using CRISPR-mediated genome editing. We recovered three independent alleles, all of which result in grossly wild-type animals that exhibit no apparent vulval defects (Fig. 1). Specifically, unlike *let-60(n1046[G13E]),* the substitution does not confer the Muv phenotype (Fig. 1A-C,G). Further, in all larval *let-60(gu287[G13D])* mutants evaluated, only three vulval precursor cells divided to produce vulval tissue as seen in wild type, and animals exhibit a vulva with typical morphology in the fourth larval stage (Fig. 1D-F,H). By contrast, in *let-60(n1046[G13E])* mutants, between three and six cells divide – resulting in a normal vulval structure and in up to three other clusters of cells that form small vulva-like structures in animals at larval stage 4 (L4) – and undergo morphogenesis to produce bumps of tissue in adults. We conclude that, unlike the G13E substitution, substitution of G13D did not sufficiently disrupt LET-60 function to produce an activated Ras phenotype.

### A MosSCI-based method to assess oncogenic Ras variant activity in *C. elegans*

We next aimed to test the activity of other oncogenic variants in the *C. elegans* model. After preliminary efforts to introduce alleles directly into the *let-60* locus suggested many such alleles confer a lethal phenotype (not shown), we sought an alternate approach that would permit functional assessment of multiple variants in the vulval development model, where alterations to Ras activity (either activated and disrupted) confer specific outcomes that can be easily identified visually by using a dissecting microscope or similar optical methods. We utilized the MosSCI method of Mos1 transposon-mediated single-copy insertion to introduce one additional copy of the *let-60* gene into a defined landing site in the *C. elegans* genome (Frøkjær-Jensen et al., 2014) that comprised 4 kb genomic DNA, including the *let-60* coding exons as well as 1 kb each upstream and downstream, and including all of the annotated 5′ and 3′ UTR sequences. Since the inserted transposon can be made homozygous, this produced a strain with four *let-60* gene copies: two at the endogenous genomic locus and two at the landing site. For simplicity, we will refer to the endogenous locus as *let-60*, the copies at the landing site as either transposon or *Si(let-60)*, and the alleles or variants based on their impact on the predicted amino acid product. Complete genotypes, transposon names and strain nomenclature are listed in  Table S1. Insertion of single copies and expression of each was confirmed by using PCR across the insertion locus and evaluating the sequence of *let-60* cDNA derived from mRNA of each strain (Figs S2, S3).

**Fig. 2.**
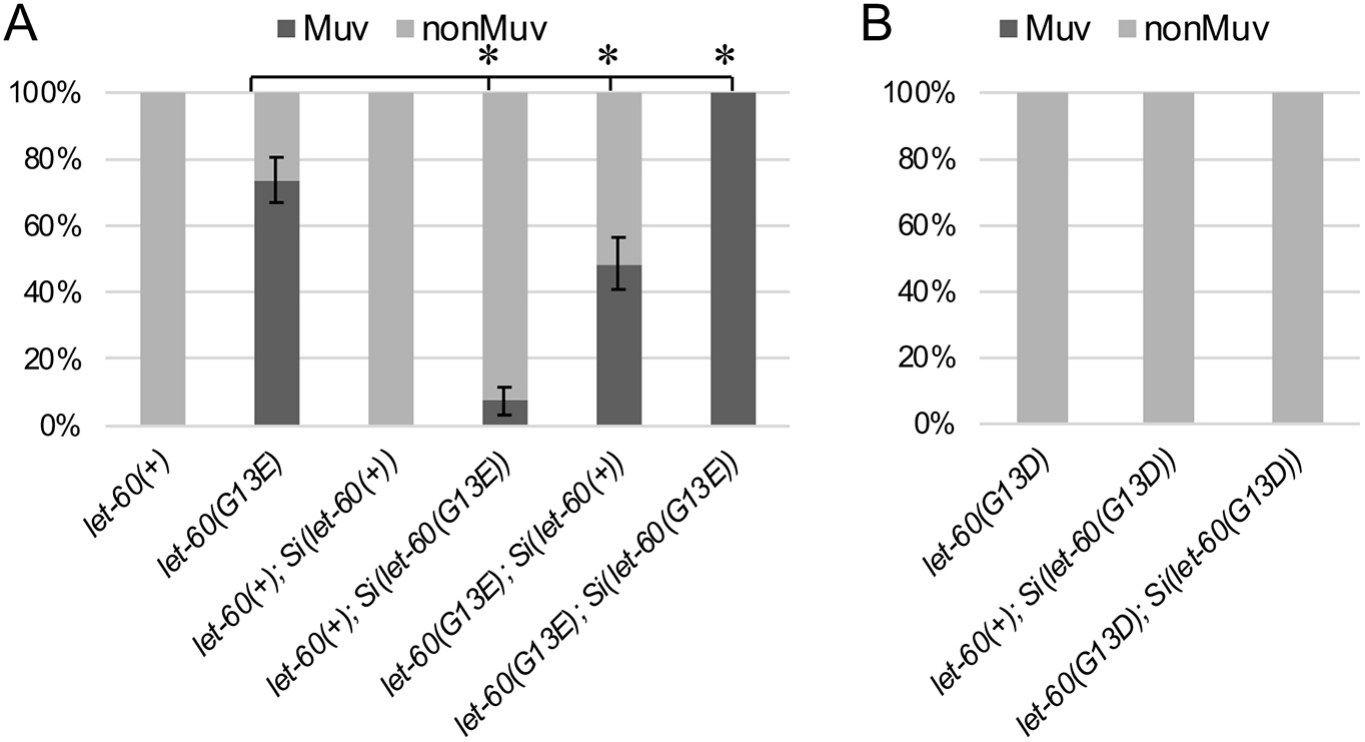
**Activity associated with *let-60* variants can be evaluated in a controlled manner using MosSCI-based transgenes.** (A). Animals homozygous for a *let-60* gene copy at the ttTi5605 landing site on LG II – i.e. *let-60(+); Si(let-60(+))* – and corresponding to four rather than the wild-type two gene copies in the genome, exhibit a wild-type phenotype. Introduction of the *let-60(G13E)* variant at this landing site can weakly interfere with wild type, producing a low frequency of Muv animals. Likewise, transposon-introduced wild-type gene copies can modulate *let-60(G13E)* at the endogenous locus, reducing the frequency of Muv animals. Finally, presence of *let-60(G13E)* at all four sites (endogenous and transposon site) confers a strong Muv phenotype. Sample sizes: *n*=40 for *let-60(+)*; *n*=42 for *let-60(G13E)*; *n*=44 for *let-60(+); Si(let-60(+))*; *n*=40 for *let-60(+); Si(let-60(G13E))*; *n*=41 for *let-60(G13E); Si(let-60(+))*; *n*=42 for *let-60(G13E); Si(let-60(G13E))*. (B) In contrast to *let-60(G13E)*, increasing the dosage of *let-60(G13D)* by adding transposon-based copies at *ttTi5605* does not alter the phenotype. Sample sizes: *n*=43 for *let-60(G13D)*; *n*=45 for *let-60(+); Si(let-60(G13D))*; *n*=47 for *let-60(G13D); Si(let-60(G13D))*. Error bars correspond to standard error of the proportion. Asterisks in A indicate statistical differences (*P*<0.05); two proportion *Z*-test and Bonferroni correction, comparing each G13E-containing strain to *let-60(G13E)* (control).

When a wild-type copy of *let-60* was introduced on the transposon, the animals are grossly normal and exhibit no Muv phenotype (Fig. 2A). This is in contrast to the case where multiple copies of the gene are introduced as part of an extrachromosomal array (Han and Sternberg, 1990). Introduction of a *let-60(G13E)* transposon caused a weak Muv phenotype, whereas introduction of wild-type *Si(let-60(+))* into the *let-60(G13E)* background produced an intermediate phenotype. This demonstrates that the transposon-based gene copies produced a product that can influence vulval development. Since the Muv phenotype of these two genotypes was not identical, the activity of the transposon-based gene appeared lower than that at the endogenous locus, a phenotypic observation consistent with the relative abundance of the transgene-based variant in mRNA (Fig. S3). Finally, inclusion of the G13E variant at both loci enhanced the phenotype. Together, these results indicate that animals were sensitive to dosage of the G13E variant and that the effect does not dependent on the presence of the wild-type gene copy. However, for this variant, wild-type protein did compete with mutant to promote a more wild-type response in animals that express both. To ask whether the G13D variant exhibits a similar dosage effect, we produced an *Si(let-60(G13D))* transgene and introduced it into the *let-60(gu287[G13D])* background (Fig. 2B). These animals remained phenotypically wild type, indicating that increasing the dosage of this variant does not alter the effect observed in the mutant.

### *C. elegans* vulval development exhibits distinct responses to different disease-associated Ras variants

**Fig. 3.**
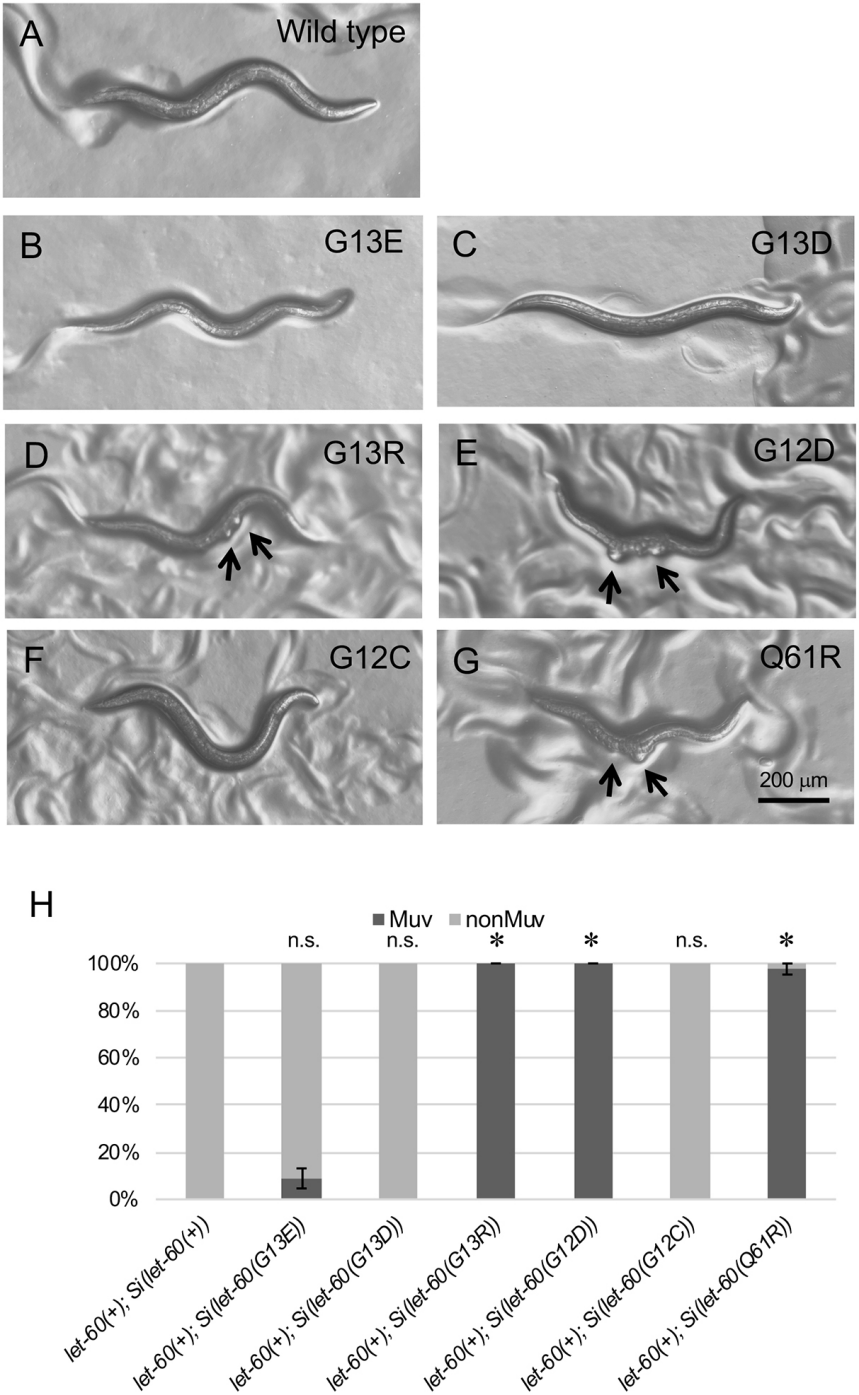
**Introduction of some mutations orthologous to human oncogenic Ras variants can produce the Muv phenotype in *C. elegans***. Adult animals homozygous for transposons bearing wild-type or different *let-60* oncogenic variants. (A-G) Animals in each panel are oriented as in Fig. 1, with their anterior to the right and ventral down. Animals homozygous for wild-type (A)*, let-60(G13D)* (C) or *let-60(G12C)* (F) transposons exhibit a smooth ventral surface. Most *let-60(G13E)* (B) transposon-bearing animals are similarly nonMuv. In contrast, the Muv phenotype is observed among animals bearing the *let-60(G13R)* (D), *let-60(G12D)* (E) or *let-60(Q61R)* (G) variants. Ventral bumps indicative the Muv phenotype are indicated with black arrows. (H) Quantification of the Muv phenotype. Sample sizes*: n*=53 for *let-60(+); Si(let-60(+))*; *n*=45 for *let-60(+); Si(let-60(G13E)*; *n*=41 for *let-60(+); Si(let-60(G13D))*; *n*=43 for *let-60(+); Si(let-60(G13R))*; *n*=37 for *let-60(+); Si(let-60(G12D))*; *n*=37 for *let-60(+); Si(let-60(G12C)*; *n*=42 for *let-60(+); Si(let-60(Q61R))*. Error bars indicate the standard error (±s.e.) of the proportion. Asterisks indicate statistical differences (*P*<0.05); two proportion *Z*-test and Bonferroni correction, comparing each single-copy oncogenic variant transposon to the wild-type transposon. n.s., not significant.

**Fig. 4.**
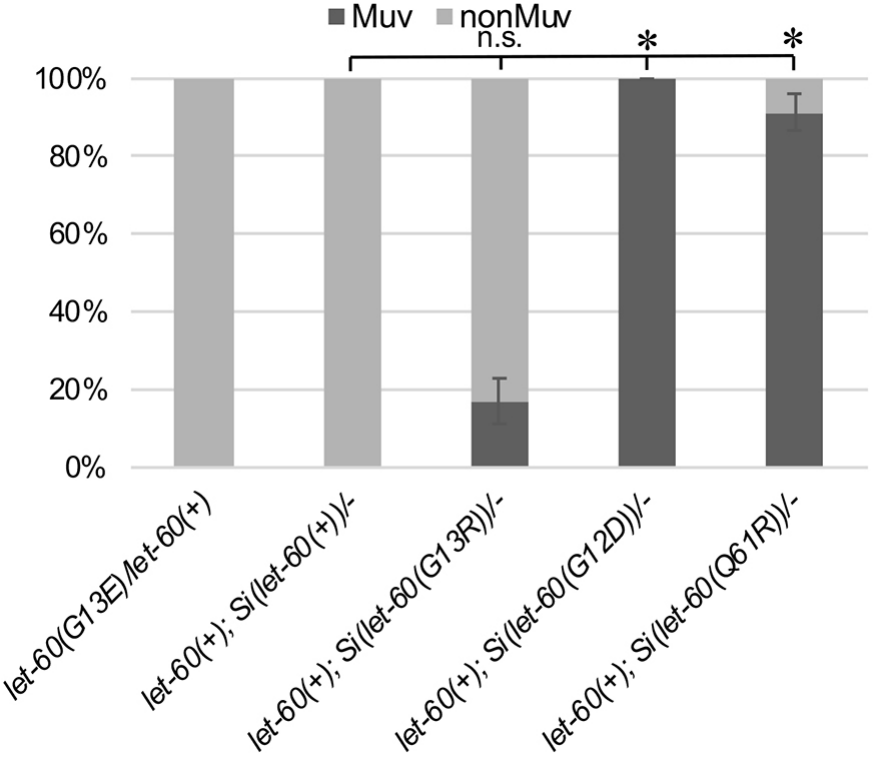
***let-60* oncogenic variants differ in dosage sensitivity.** A single copy of the *let-60(G12D)* or *let-60(Q61R)* transposon (in an otherwise wild-type background) is sufficient to produce a strong Muv phenotype, whereas *let-60(G13R)* produces only a weak Muv phenotype. The dominance test for *let-60(n1046[G13E])* was completed in parallel, as a comparison. Sample sizes: *n*=30 for *let-60(G13E)/let-60(+)*; *n*=31 for *let-60(+); Si(let-60(+))/−*; *n*=41 for *let-60(+); Si(let-60(G13R))/−*; *n*=32 for *let-60(+); Si(let-60(G12D))/−*; *n*=35 for *let-60(+): Si(let-60(Q61R))/−*. Error bars indicate the standard error (±s.e.) of the proportion. Asterisks indicate statistical differences (*P*<0.05); two proportion *Z*-test and Bonferroni correction, comparing each single-copy oncogenic variant transposon to the wild-type transposon. n.s., not significant.

We utilized the MosSCI method to introduce *let-60* transposons encoding a set of additional oncogenic substitution mutations prevalent in human cancer samples, including G13R, G12D, G12C and Q61R (Fig. 3). Data for a G13S transgene that was also produced and analyzed but that exhibits higher expression, and may represent insertion of multiple gene copies, are included in  Fig. S4. Like *Si(let-60(G13D))*, *Si(let-60(G12C))* caused no gross defects to vulva development (Fig. 3C,F), whereas *Si(let-60(G13R)), Si(let-60(G12D))* and *Si(let-60(Q61R))* conferred a strong Muv phenotype on all or most animals (Fig. 3D,E,G). We next asked whether *C. elegans* animals exhibit a dosage sensitivity to the three variants that cause a phenotype (Fig. 4). We found that − whereas a single transposon copy, rather than two as in homozygotes, is sufficient to cause a high frequency of Muv for two of the variants, i.e. *Si(let-60(G12D))* and *Si(let-60(Q61R)* – animals were sensitive to the dosage of *Si(let-60(G13R))*, and that most animals bearing only a single copy of the transgene exhibit a wild-type phenotype (Fig. 4). These results point to the capacity of each mutant variant to be subject to functional regulation and to compete with wild-type protein. *Si(let-60(G13D))* and *Si(let-60(G12C))* failed to interfere with wild type and, at least for *let-60(G13D)* (Fig. 1), essentially exhibits wild-type function. Excess wild-type protein was not sufficient to compete with *Si(let-60(G12D))* or *Si(let-60(Q61R)),* but did compete with *Si(let-60(G13R))* (Fig. 4).

**Fig. 5.**
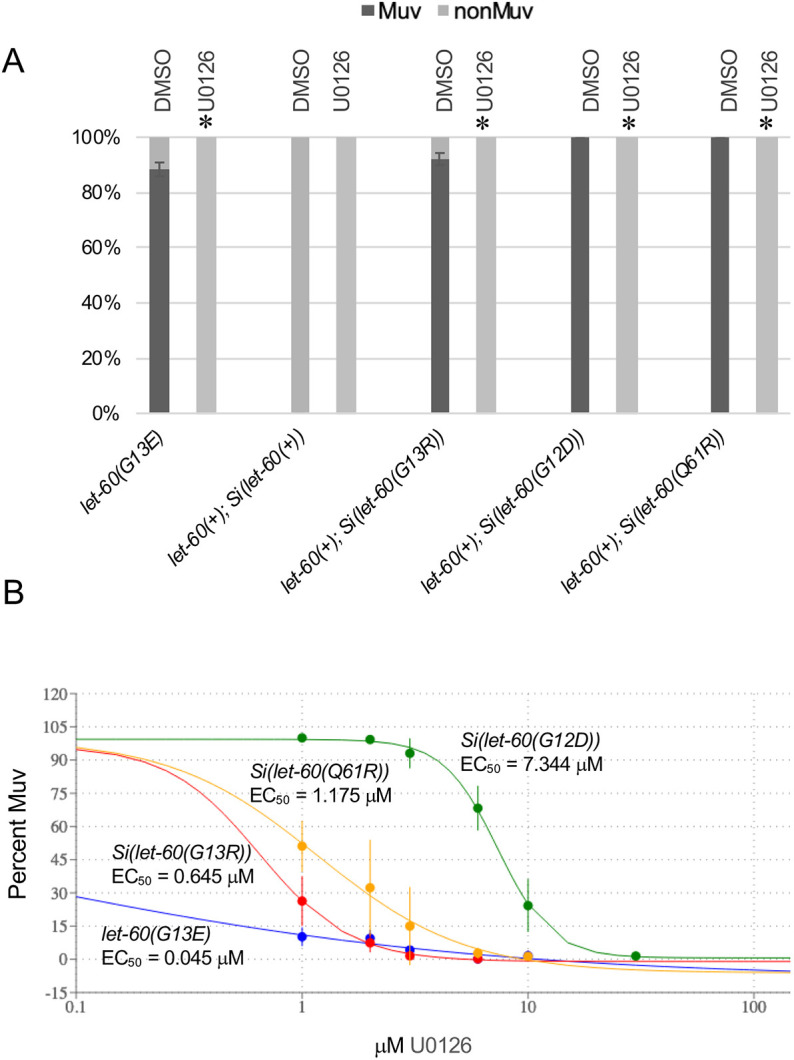
***let-60* oncogenic variants exhibit distinct dosage sensitivity to MEK activity.** (A) The Muv phenotype associated with each oncogenic variant is blocked when animals are grown on plates containing 60 µM of the MEK inhibitor U0126. Sample sizes were as follows. *let-60(G13E)*: *n*=163 (DMSO) and *n*=132 (U0126). *let-60(+); Si(let-60(+))*: *n*=139 (DMSO) and *n*=102 (U0126). *let-60(+); Si(let-60(G13R))*: *n*=152 (DMSO) and *n*=114 (U0126). *let-60(+): Si(let-60(G12D))*: *n*=54 (DMSO) and *n*= 72 (U0126). *let-60(+); Si(let-60(Q61R))*: *n*=80 (DMSO) and *n*=81 (U0126). Error bars correspond to the standard error of the proportion. Asterisks indicate statistical differences; *P*<0.05; two proportion *Z*-test and Bonferroni correction, comparing each U0126 treatment condition to its DMSO control. (B) Plotted is the response (left to right) of strains *let-60(G13E)* (blue); *let-60(+); Si(let-60(G13R))* (red); *let-60(+); Si(let-60(Q61R))* (yellow) and *let-60(+); Si(let-60(G12D))* (green) to different doses of the MEK inhibitor U0126. Plotted are the results of eight trials, with at least 30 animals per condition per trial (exact sample sizes are included in Table S4). Pairwise comparison of EC_50_ values from eight trials for each strain was carried out using one-way ANOVA and Tukey's HSD test, indicating that all conditions are statistically different (*P*<0.05) except for *let-60(G13E)* with *let-60(+); Si(let-60(G13R))* as well as *let-60(+); Si(let-60(G13R))* with *let-60(+); Si(let-60(Q61R)).* Error bars indicate the standard error (±s.e.). The graph in B was generated using the Quest Graph calculator (AAT Bioquest, Inc.).

Finally, we evaluated the sensitivity of each variant to the MEK inhibitor U0126 to test whether the distinctions among the different oncogenic variants in *C. elegans* reflects off-target effects, or the preferential engagement by some variants with different effectors (Fig. 5). Previous genetic work in *C. elegans* has defined that, in vulval development, *let-60* acts through a canonical Ras/Raf/MEK/MPK kinase cascade (reviewed by Sternberg and Han, 1998) but that the developmental outcomes can be modulated by low ligand dosage and ‘effector switching’ to Ral/MAP4K/p38 (Zand et al., 2011). Likewise, wild-type and oncogenic human Ras proteins engage distinct effector proteins and target pathways – including Raf, Ral and PI3K – and different mutant variants are altered in their ability to engage different effectors (Céspedes et al., 2006; Hunter et al., 2015). To test how the mutant variants relate to downstream effects, we utilized the small-molecule MEK inhibitor U0126, which blocks an essential component of the canonical Ras/Raf/MEK/MPK pathway and functions in nematodes (Reiner et al., 2008). We found that, as with *let-60(n1046[G13E])*, the Muv phenotype caused by each of the oncogenic variants is sensitive to 60 μM of inhibitor (Fig. 5A), indicating that in each case the ultimate outcome is dependent on the canonical kinase signaling cascade. We next assessed each variant for sensitivity to U0126 at different doses (Fig. 5B). Although we found the EC_50_ for *Si(let-60(G12D))* to be significantly higher than that for *Si(let-60(Q61R))* and *Si(let-60(G13R))*, the response of these latter variants was statistically not different to each other. Altogether, these results show the oncogenic variants can be grouped into clear and discrete functional classes, based on whether they confer a gain-of-function phenotype, sensitivity to gene dosage and inhibition of downstream targets.

### Non-autonomous modulators of activated *C. elegans* let-60/Ras exhibit differential effects on oncogenic variants

**Fig. 6.**
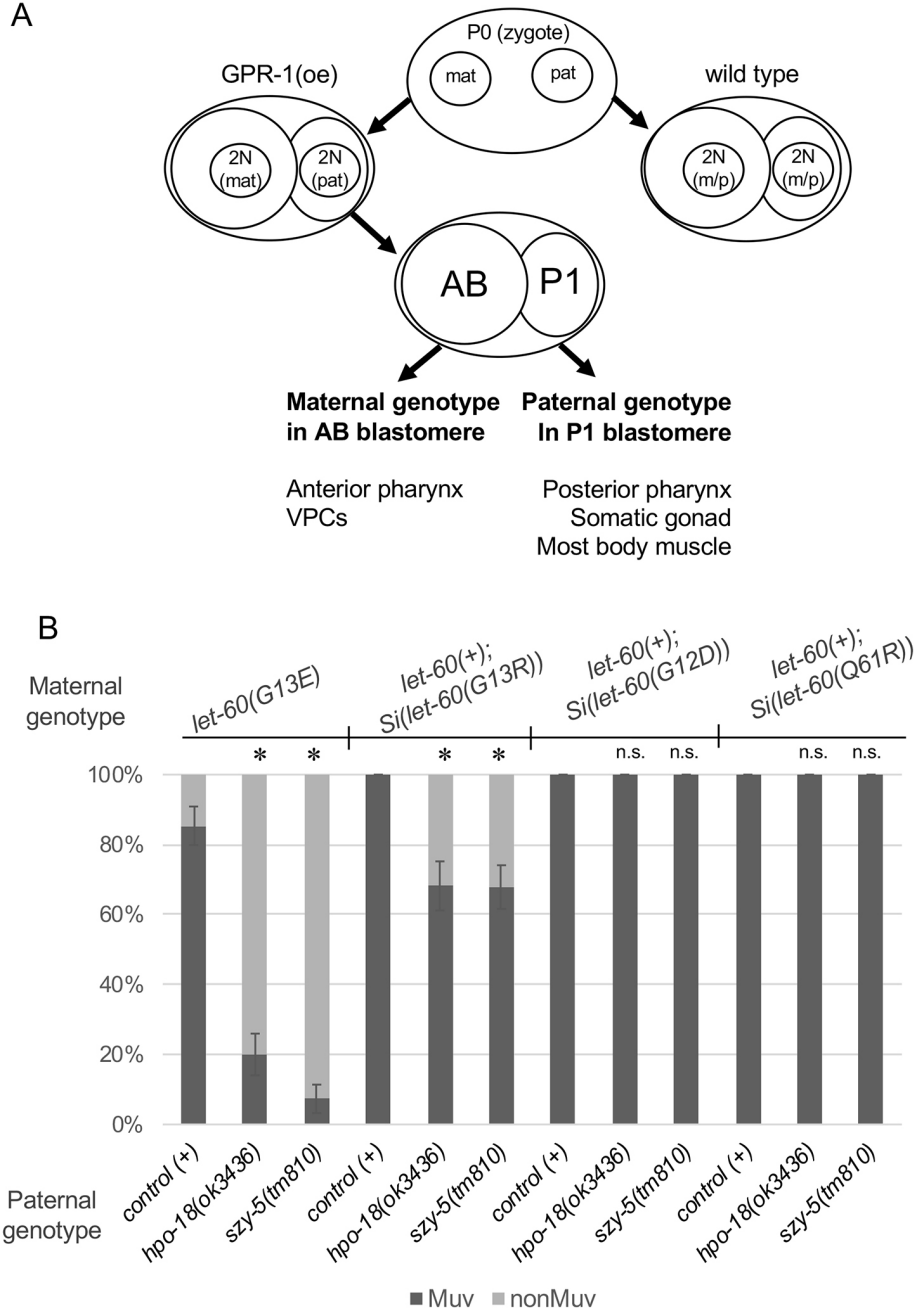
***let-60* oncogenic variants exhibit differential sensitivity to genes identified in the *let-60(G13E)* genetic background as non-autonomous modulators of activated Ras activity.** (A) Schematic representation of the genetic chimera method, as described by Artiles et al., (2019). GPR-1 is overexpressed [GPR-1(oe)] in the maternal germline and oocytes, causing the pronuclei to segregate to daughter cells without fusing in the zygote. The chimera class evaluated in this experiment is that in which replicated maternal chromosomes segregate to the AB blastomere, i.e. the precursor to the VPCs, whereas replicated paternal chromosomes segregate to the P1 blastomere, i.e. the precursor to the somatic gonad and most body wall muscle cells. (B) Chimeric animals with paternal (P1) DNA and *let-60(+)* in the P1 cell lineage, and maternal *let-60* oncogenic genotypes – with wild type for each paternal gene – in the AB cell lineage. Paternally introduced alleles of *szy-5* and *hpo-18* confer a strong suppression of the Muv phenotype in *let-60(n1046[G13E])* mutants (Corchado-Sonera et al., 2022). Animals homozygous for *Si(let-60(G13R))* transposons in AB-derived cells are moderately sensitive to disruption of *szy-5* and *hpo-18*, whereas *Si(let-60(G12D))* or *Si(let-60(Q61R))* are not. Sample sizes were as follows. *let-60(G13E)*: *n*=41 for control (+), *n*=45 for *hpo-18(ok3436)* and *n*=41 for (*szy-5(tm810)*. *let-60(+); Si(let-60(G13R)): n*=35 for control (+), *n*=44 for *hpo-18(ok3436)* and *n*=56 for *szy-5(tm810)*. *let-60(+); Si(let-60(G12D)): n*=32 for control (+), *n*=30 for *hpo-18(ok3436)* and *n*=33 for *szy-5(tm810)*; *let-60(+); Si(let-60(Q61R))*: *n*=50 for control (+), *n*=30 for *hpo-18(ok3436)* and *n*=36 for *szy-5(tm810)*. Error bars indicate the standard error (±s.e.) of the proportion. Asterisks indicate statistical differences (*P*<0.05); two proportion *Z*-test and Bonferroni correction, comparing each variant with mutant paternal contribution to its wild-type control. n.s., not significant.

In a previous genetic screen, we have identified a set of non-autonomous modulators of activated *let-60* (Corchado-Sonera et al., 2022). These genes, specifically when knocked down in mesodermal tissues, revert the Muv phenotype of animals bearing the genotype *let-60(n1046[G13E])* to a more wild-type one. We did also find two genes, *hpo-18* (encoding ATP synthase F1ε) and *szy-5* (encoding a zinc finger protein), that mediate this effect in genetically chimeric animals carrying the *let-60(n1046[G13E])* mutation only in cells derived from the embryonic precursor AB (including VPCs), and the mutant allele of each modifying gene only in cells derived from the embryonic precursor P1 (including anchor cell, somatic gonad and most body-wall muscles). To ask whether these modifiers are specific to *let-60(n1046[G13E])* or whether they impact the activity of other mutant variants, we used the same experimental framework in this current study to evaluate the sensitivity of the transposon variants that cause the Muv phenotype to the non-autonomous modulators (Fig. 6A). First, we observed that inclusion of the oncogenic genotype only in cells derived from the AB precursor is sufficient to cause the Muv phenotype, as is the case for *let-60(n1046[G13E])* (Fig. 6B). Next, we evaluated the sensitivity of each variant to mutation of *hpo-18* or *szy-5*. We found that *let-60(G12D)* and *let-60(Q61R)* are insensitive to disruption of either gene in P1-derived cells. *let-60(G13R)* exhibits sensitivity, but to a lesser extent than that observed with *let-60(n1046[G13E])*. We concluded that the modifiers are variant- rather than gene-specific in their effect and, preferentially, alter the phenotype of variants with a weaker impact on pathway activation.

## DISCUSSION

In this study we evaluated a set of oncogenic variants for their function in *C. elegans* by using CRISPR-mediated genome editing and controlled single-copy insertion methods. Aiming to classify and uncover unique sensitivities for the different variants, we found they fall into discrete groups (Table 1). G13D and G12C failed to disrupt signaling sufficiently to cause a Muv phenotype. G13R, Q61R and G12D exhibited distinctions based on their sensitivity to gene dosage, MEK inhibition and genetic modifiers. While Q61R is more similar to G13R regarding the sensitivity to MEK inhibition and to G12D regarding sensitivity to the dosage and genetic modifier, it is noticeable that the mutant variants can be roughly classified according to ‘strength’ of the phenotypic effect. We anticipate that other morphological and molecular readouts (Burdine et al., 1998; de la Cova et al., 2017) may identify signaling perturbations in animals with a grossly wild-type phenotype, as well as more quantitative distinctions among each of the tested variants, allowing further classification. Importantly, however, even when using a simple measure reflecting the gross morphological and simple qualitative (Muv/nonMuv) consequences of these mutations, clear functional distinctions are apparent. We, therefore, hypothesize that mutations, such as *let-60(gu287[G13D]) –* while they confer no overt phenotype on their own – may provide a sensitized genetic background for enhancement screening, just as the *let-60(n1046[G13E])* background is amenable to suppressor screening. Mutants disrupted for *gap-1* – which encodes one of two GAP proteins that negatively regulate LET-60/Ras activity in the vulval cells – have, similarly, provided a sensitized background for identification of genetic enhancers (Liu et al., 2017; Stetak et al., 2008). We conclude that distinct features of different Ras variants can be uncovered by using the *C. elegans* vulval development system, and by using simple morphological criteria that are easily adapted to moderate and high throughput screening.

**Table 1.**
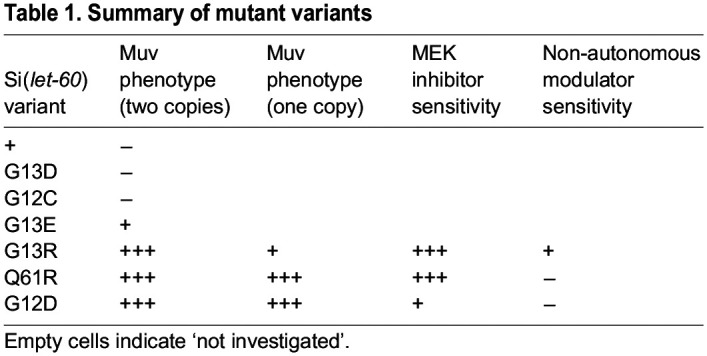
Summary of mutant variants

Our experiments identified features of Ras biology suitable to model by using *C. elegans* and defined how oncogenic variants differentially impact them. Although Ras alters developmental-fate choices in vulval development – rather than cell cycle progression directly – it provides a sensitive *in vivo* assay. Indeed, genetic studies in *C. elegans* have been crucial in establishing the order in which proteins act with Ras, and in identifying many novel but conserved proteins that influence Ras activity (reviewed by Sternberg and Han, 1998). For this current study, we chose to introduce human oncogenic variants into the *C. elegans* gene – rather than to ectopically express a human protein (Das et al., 2021) – to ensure species-typical gene regulation and that engagement with effector proteins retains any co-evolutionary relationships. In this context, we compared our variant-specific results to studies using human proteins. Specifically, we noticed that the two variants that fail to confer the Muv phenotype (G12C and G13D) exhibit relatively more wild-type behavior in an assay of intrinsic GTPase activity and a quantitative assay of RAF affinity (Hunter et al., 2015). We interpret that, in the *C. elegans* system, proteins capable of engaging the canonical kinase pathway (via the standard interaction with RAF) and regulation via intrinsic GTPase activity behave in a sufficiently wild-type manner, so that normal development proceeds. In contrast, G12D and Q61R exhibit similar dosage-insensitive properties in our assays but differ with respect to their sensitivity to U0126-induced inhibition of MEK. Biochemically, Q61R has noticeably compromised GTPase activity (Burd et al., 2014), consistent with a direct role in catalysis for Q61 (Buhrman et al., 2010), whereas G12D retains an intrinsic GTPase activity comparable to G12C and G13D (Hunter et al., 2015). KRAS variants with a bulky side chain at G12 – including G12D – exhibit lower affinity for RAF (Hunter et al., 2015); moreover, experimentally, mammalian G12D mutants preferentially engage alternative downstream pathways, such as PI3K or Ral (Céspedes et al., 2006). We hypothesize that these differences may underlie the phenotypic distinctions between G12D and Q61R in our assays. A Ral-mediated response to Ras has been shown to participate in *C. elegans* vulval development (Shin et al., 2019; Zand et al., 2011) and, although the activity of G12D ultimately depends on the canonical cascade, differential engagement of this alternative pathway might contribute to the phenotypic distinctions we observed at low inhibitor dose. Future experiments using genetic mutants and more quantitative readouts will be important in dissecting the altered cellular consequences of each mutant.

A key distinction among the Ras variants is the sensitivity of animals to both dosage of the mutant gene and inhibition of the downstream kinase MEK. The *let-60(n1046[G13E])* allele is dosage sensitive, as it is only weakly semi-dominant (Fig. 4) (see also Han and Sternberg, 1990) and animals bearing four copies have a stronger Muv phenotype than those bearing two (Fig. 2). Likewise, we found dosage sensitivity for *Si(let-60(G13R))* but not for *Si(let-60(G12D))* or *Si(let-60(Q61R)).* This sensitivity might reflect differences in the capacity of wild-type proteins from the endogenous *let-60(+)* to interfere with or impact the mutant variant activity within active Ras multimers or nanoclusters (reviewed by Simanshu et al., 2023). Alternatively, the results might reflect the importance of expression level on oncogenic ‘strength’ of a particular mutant variant. Many studies that evaluate differences among oncogenic variants (including this one) utilize techniques to control expression levels and other features across conditions to better focus on the differences in protein activity. However, protein abundance, whether influenced by transcription, translation or stability, is a critical component that influences the phenotypic outcome of different mutations in human and mouse cells. For example, differences in transcription levels determine whether a particular mutation causes tumor formation or apoptosis (Sarkisian et al., 2007), and translation codon representation (affecting translation rate) can affect the tumorigenicity of mutants with identical transcriptional characteristics (Lampson et al., 2013). Experimentally, a wide range of mutations can be ‘oncogenic’ in cells that are not found in human disease samples, and these results are interpreted to relate to a ‘sweet-spot’ between oncogenic Ras dosage and dysregulation to yield a disease outcome (Hidalgo et al., 2022; Hood et al., 2023; Li et al., 2018). This sweet-spot, however, arises from the complex interaction of protein abundance, oncogenic variant and cellular context (Le Roux et al., 2022). In *C. elegans*, inputs that modulate protein abundance have, likewise, been shown to reduce the phenotypic effect of the *let-60(G13E)* variant (Kramer-Drauberg et al., 2020). Our results demonstrate that the vulval development process is highly sensitive to the effects of *Ras* dosage and mutant variant, and can be used to dissect these fundamental inputs and the cellular processes that modulate them.

## MATERIALS AND METHODS

### Worm maintenance and genetics

*Caenorhabditis elegans* strains were grown on NGM plates seeded with *Escherichia coli* strain OP50 as a food source (Stiernagle, 2006). Strains were grown and experiments performed at 20°C, unless stated otherwise. The wild-type *C. elegans* used was strain N2 Bristol. Specific strains and genotypes are listed in  Table S1. All strains not deposited with the *Caenorhabditis* Genetics Center are available upon request.

### Production of *let-60(G13D)* mutant alleles

Three *let-60(G13D)* mutant alleles, i.e. *let-60(gu282)*, *let-60(gu283)* and *let-60(gu287)*, were generated using the method described by Arribere et al., (2014). Single-guide RNA (sgRNA) targeting the *let-60* genomic locus was introduced into plasmid pDD162 (Dickinson et al., 2015), yielding plasmid pBP1 (for primers and plasmids see Tables S2 and S3). pBP1 (50 ng/μl) was then injected into the germline of wild-type *C. elegans* hermaphrodites together with a single-stranded DNA-repair template (20 ng/μl) and a fluorescent reporter (*myo-2::mCherry*, pCFJ90, 25 ng/μl) as a transformation marker (for primers and sequences, see Table S2). mCherry-positive F1 animals were selected and allowed to self-cross. After offspring on each plate founded by an F1 parent depleted the bacteria, a portion was saved and the remainder prepared to recover genomic DNA. Genomic DNA was used as template for PCR. The PCR product was then subjected to Sanger sequencing to identify plates founded by – typically heterozygous – candidate mutants. Animals were recovered from these candidate heterozygous plates, individually plated and allowed to self-cross. Plates containing their resulting offspring (F2) plates were also screened via Sanger sequencing to identify homozygous strains.

### Plasmids and single-copy insertion transgene production and validation

A 4.1 kb genomic fragment containing all transcribed regions and the 1 kb upstream sequence of *let-60* was produced using Phusion DNA polymerase (NEB) and wild-type genomic DNA template, and assembled into the XhoI site of plasmid pCFJ151 (Frøkjær-Jensen, 2015), which contains a selectable marker, i.e. *Cbr-unc-119(+)*, and flanking sequences to allow MosSCI insertions at ttTi5605. Sequences used for primer and plasmid design were downloaded from Wormbase (Davis et al., 2022). The insert was confirmed by restriction enzyme digest and Sanger sequencing (yielding plasmid pLK24). While it is not known if this sequence includes all important *cis* regulatory elements for *let-60*, our transgene experiments (Figs 2, 3) and sequence of cDNA from L3 animals (Fig. S3) demonstrate that the transposon-based gene is expressed and can impact vulva development. Mutant variants were introduced into this template, using PCR-based site-directed mutagenesis (Bachman, 2013). Mutations were confirmed with Sanger sequencing. All plasmids are available upon request.

Injection mixes [each *let-60* plasmid produced above (25 ng/μl; MosI transposase-encoding plasmid pCFJ601 (50 ng/μl; Addgene plasmid #34874) and pCFJ90 (*myo-2::mCherry* (20 ng/μl; Addgene plasmid #19327) encoding a marker to exclude extrachromosomal arrays)] were injected into *C. elegans* strain EG4322 (WormBase ID: WBStrain00006687; https://wormbase.org/species/c_elegans/strain/WBStrain00006687#03--10 ), which contains the ttTi5605 landing site on LG II and *unc-119(ed9)*. NonUnc F1s were plated individually. Those that segregated nonUnc offspring that were negative for mCherry fluorescence were considered further, and offspring were plated individually to establish homozygous lines that segregate nonUnc offspring. These lines were screened using PCR to ensure homozygosity and transgene insertion at the landing site (Fig. S2), followed by PCR and sequencing of the *let-60* copy in the landing site (using transgene-specific primers,  Table S2), to ensure retention of the intended mutation.

Plasmids corresponding to wild type, G13E or 12 different oncogenic variants were initially produced. All plasmids were injected as above, to produce at least 50 F1 nonUnc offspring. While one or more insertions corresponding to the variants reported here – i.e. wild type, G13D, G13R, G12D, G12C and Q61R, as well as G12S as a potential multi-copy insertion (Fig. S4) – were obtained, no insertion lines were produced with plasmids encoding G12 V, G12A, G12R, G13C, Q61 L or Q61 K. While additional trials might yield such lines, the presence of many sterile and sickly animals among nonUnc F1 offspring following these injections suggests that these variants cannot produce fertile strains, even as single copy in the MosSCI landing site, and that alternative methods, such as using conditional and/or cell-specific expression, are necessary to evaluate the function of these variants in the *C. elegans* vulval development system.

Expression of the introduced transgene (Fig. S3) was evaluated by harvesting RNA from synchronized L3 animals (Trizol), production of first strand cDNA using random primers (Superscript III), followed by PCR and Sanger sequencing. Since the transgene sequences incorporate genomic DNA, the primers amplify cDNA derived from RNA produced by both the endogenous locus and the transgene. Primers (Table S2) were selected to ensure that the sequence reads were produced from spliced cDNA, rather than genomic DNA.

### Microscopy and phenotypic analysis

The multivulva (Muv) phenotype (Figs 1–4,  Fig. S4) was assessed by selecting hermaphrodite animals at L4 produced from well-fed parents. These L4 animals were aged overnight, and scored for presence of ectopic vulval protrusions the next day. Animals of heterozygous or single transgene genotype (Fig. 4) were generated by crossing homozygous hermaphrodites with males from strain FX17749, which contains the balancer *hT2* that was dominantly marked with *myo-2::gfp* (*qIs48*). GFP-positive hermaphrodite F1 animals at L4 were selected for scoring. Animals were assessed for MEK sensitivity and dose response (Fig. 5) by treatment with U0126 as in (Dawes et al., 2017). Briefly, L1 animals were plated onto 35 mm NGM plates spread with either MEK inhibitor U0126 (AdooQ Bioscience) or DMSO (solvent control) in M9 buffer (Stiernagle, 2006) and seeded with OP50 1 day prior to plating. Animals were allowed to mature for 2–3 days before they were scored for the Muv phenotype. Inhibitor concentration is reported as the calculated concentration using the full volume of NGM in the plate. Vulval cell development and induction (Fig. 1) were evaluated in animals at L4 using DIC optics at 100× magnification as in (Chamberlin et al., 2020). EC_50_ values were calculated and displayed in Fig. 5B using the Quest Graph calculator (AAT Bioquest, Inc., https://www.aatbio.com/tools/ec50-calculator).

### Production of genetic chimeras

Genetic chimeras (Fig. 6,  Fig. S4) were generated as described by Artiles et al. (2019) and Corchado-Sonera et al. (2022). *let-60* transposon strains were crossed with strains bearing transgene *ccTi1594* – which causes overexpression of GPR-1 in the germline, yielding segregation of maternal and paternal DNA into separate blastomeres in the zygote – to produce maternal strains CM2783, CM2982, CM3042 and CM3043 (for strain details see Table S1). Hermaphrodites from these strains were crossed with control (N2) males or heterozygous mutant males, i.e. *szy-5(tm810)/hT2(qIs48)* or *hpo-18(ok3436)/nT1(qIs51)*, bearing deletion alleles of genes that had previously been shown to revert the Muv phenotype associated with *let-60(n1046[G13E])* when disrupted in P1-derived (non-VPC) cells (Corchado-Sonera et al., 2022). Chimeric offspring in which the maternal genome segregates into AB (and paternal into P1) exhibit a characteristic pattern of fluorescence (mCherry from *hjSi20*) in the anterior pharynx that is easy to identify under a dissecting microscope (Artiles et al., 2019). mCherry-positive chimeric offspring that lacked GFP – i.e. indicating animals that lack the paternal balancer chromosome *hT2(qIs48)* or *nT1(qIs51)* – were selected at larval stage 4 (L4) and allowed to mature to adulthood to evaluate the vulva phenotype.

### Sequence analysis

Amino acid sequences used in  Fig. S1 were obtained from NCBI (LET-60, NP_502213.3) or UniProt (KRAS, P01116-1; HRAS, P01112-1; NRAS, P01111) and aligned using ClustalW (Thompson et al., 1994). The variant frequencies in human cancer samples were obtained from the GDC data portal (Data Release 38.0) (Grossman et al., 2016). All variants of *KRAS*, *HRAS* and *NRAS* were summarized from all cancer types across all GDC datasets. These data disproportionately reflect *KRAS* variants as 67% of the total mutations identified to affect this gene.

### Statistics

A threshold of α=0.05 was selected for all statistical tests. Proportional data (percent Muv) were evaluated using a two sample proportions, two-tailed *Z*-test (using the calculator at https://www.socscistatistics.com/tests/ztest/), with a standard (α/*k*) Bonferroni correction for multiple tests where appropriate. Pairwise comparison of EC50 values in Fig. 5B was evaluated using one-way ANOVA and Tukey's honest significance (HS) test (using the calculator at https://www.socscistatistics.com/tests/anova/default2.aspx) A two sample, two-tailed *t*-test was used for the data in Figure 1H (using the *t*-test function within Excel).

## Supplementary Material

10.1242/dmm.050577_sup1 Supplementary information

Table S1. Strains used in Lyu and Chamberlin

Table S2. Primers used in Lyu and Chamberlin

Table S3. Plasmids used in Lyu and Chamberlin

Table S4. Sample sizes for data in Fig. 5B in Lyu and Chamberlin
