# Supplementary material for: Functional distinction in oncogenic Ras variant activity in Caenorhabditis elegans
Source: Dis Model Mech. 2024 Aug 14;17(8):dmm050577. doi: 10.1242/dmm.050577 (PMC11340813; doi:10.1242/dmm.050577)
Supplement: Supplementary information [file dmm-17-050577-s1.pdf]

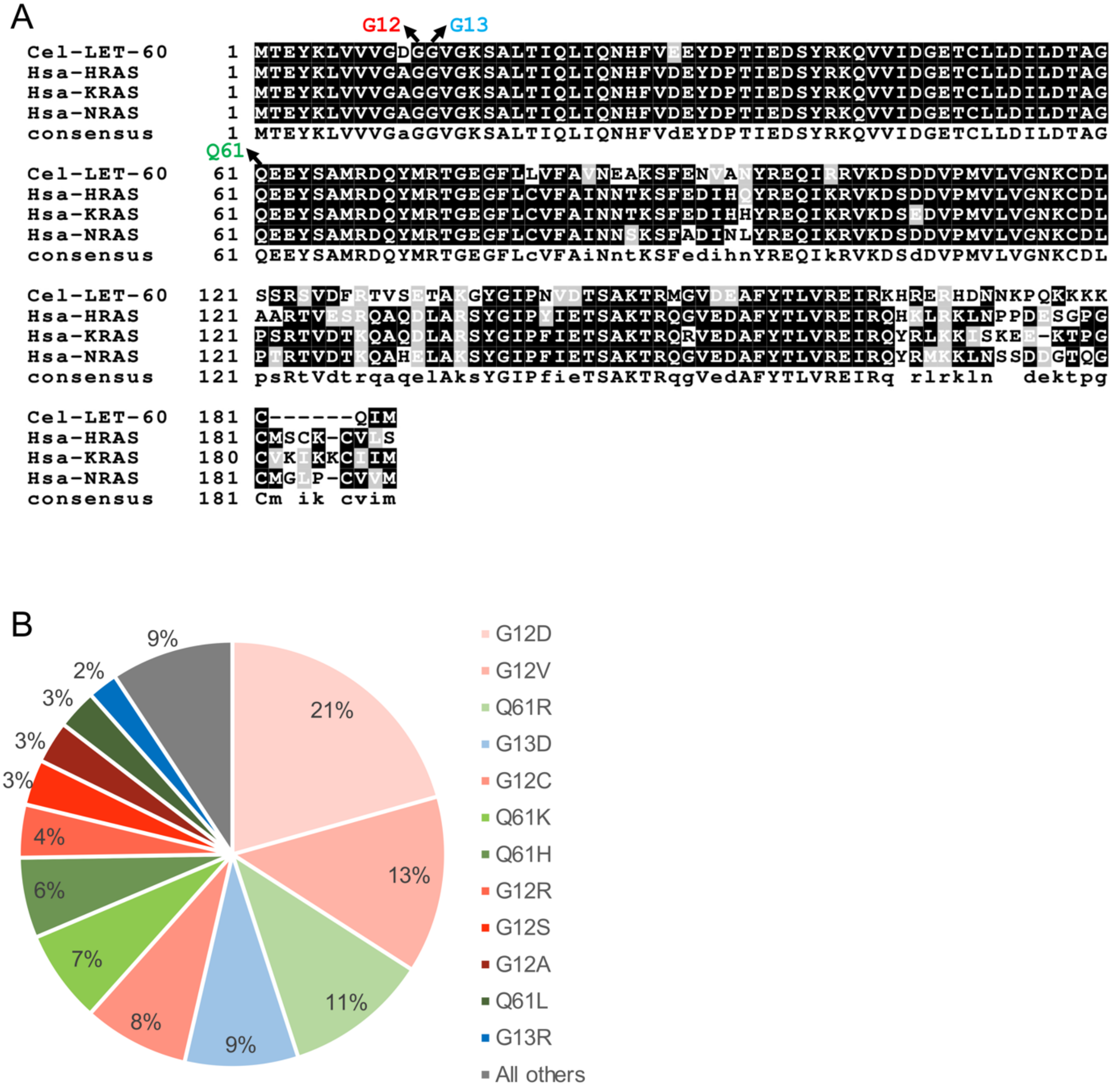

Fig. S1. Protein features of *C. elegans* LET-60 and related human Ras proteins. A. Amino Acid alignment of *C. elegans* and human Ras proteins. The *C. elegans* protein exhibits 75-77% identity with the human proteins, and the position and identity of amino acids commonly altered in human cancer (G12, G13, Q61) are conserved. B. Frequency of Ras mutant variants present in patient samples in the NCI GDC database (Grossman et al., 2016). All variants identified in any cancer type from any of the three *Ras* genes are included. Data disproportionately reflect variants in *KRAS*, as 67% of the total mutations identified affect this gene.

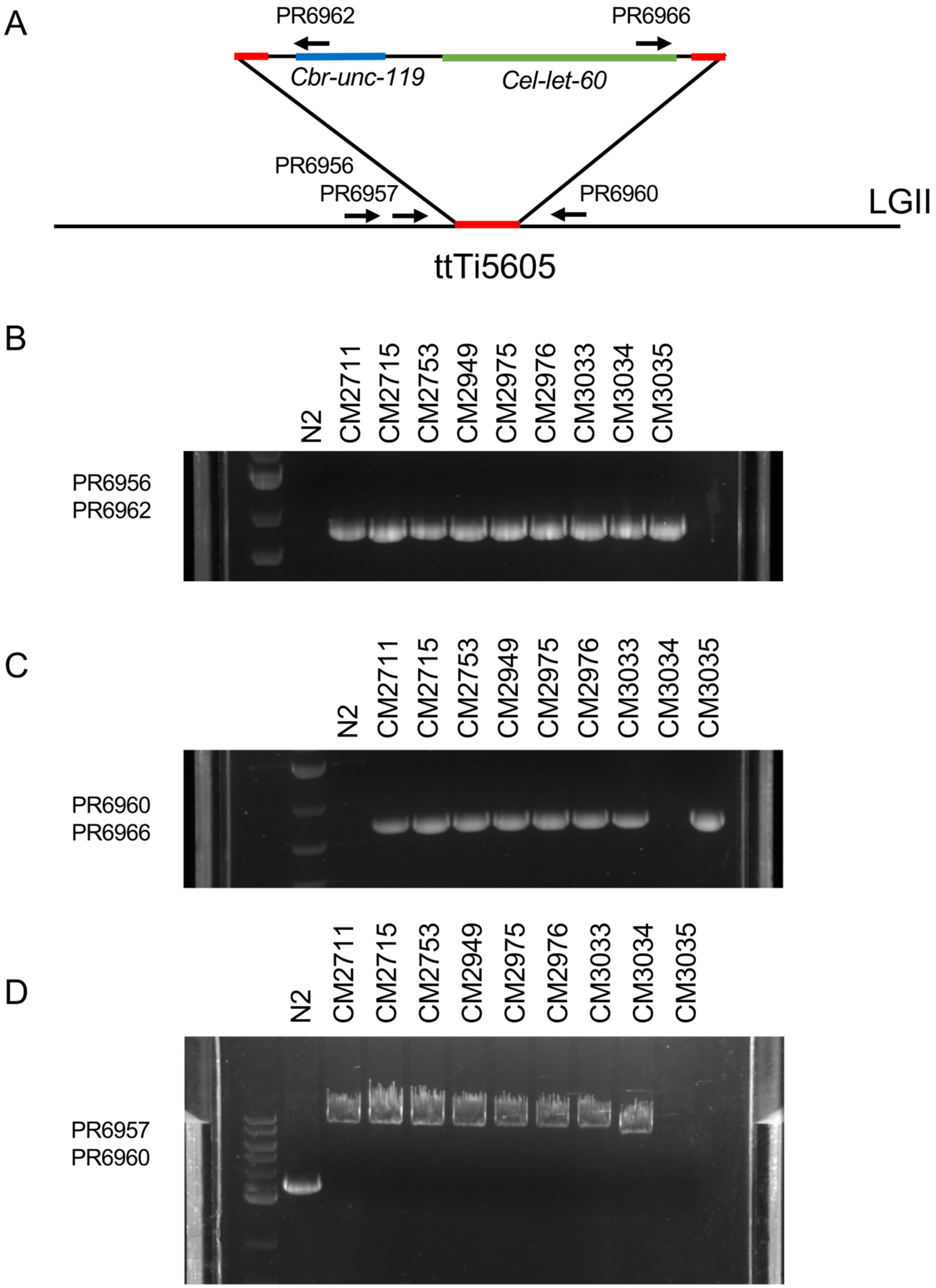

Disease Models & Mechanisms • Supplementary information

Fig. S2. Characterization of transposon insertion lines. A. Schematic representation of *let-60* insertions at the ttTi5605 landing site on LGII and primers used. B. Product using primers PR6956 and PR6962 on genomic DNA template from candidate insertion strains. Insertion strains yield a product, but control strains do not. C. Product using primers PR6960 and PR6966 on genomic DNA template from candidate insertion strains. Insertion strains yield a product, but control strains do not. D. Product using primers PR6957 and PR6960 on genomic DNA template from candidate insertion strains. Single insertion strains yield a large (>10 kb) product compared to control. DNA from strain CM3034 failed to produce a product with PR6960 and PR6966, and yields a shorter product with PR6957 and PR6960, suggesting a deleted or incomplete product was inserted on the chromosome. This strain was not considered further. While the left and right joins for strain CM3035 are correct, CM3035 DNA failed to produce a product with PR6957 and PR6960. This, in combination with the variant representation in cDNA (Supplemental Figure 3), suggests more than *one let-60(G12S)* gene copy may be inserted. Consequently, while data were collected for this strain in parallel to the others, we have separated them from the main text into Supplemental Figure 4.

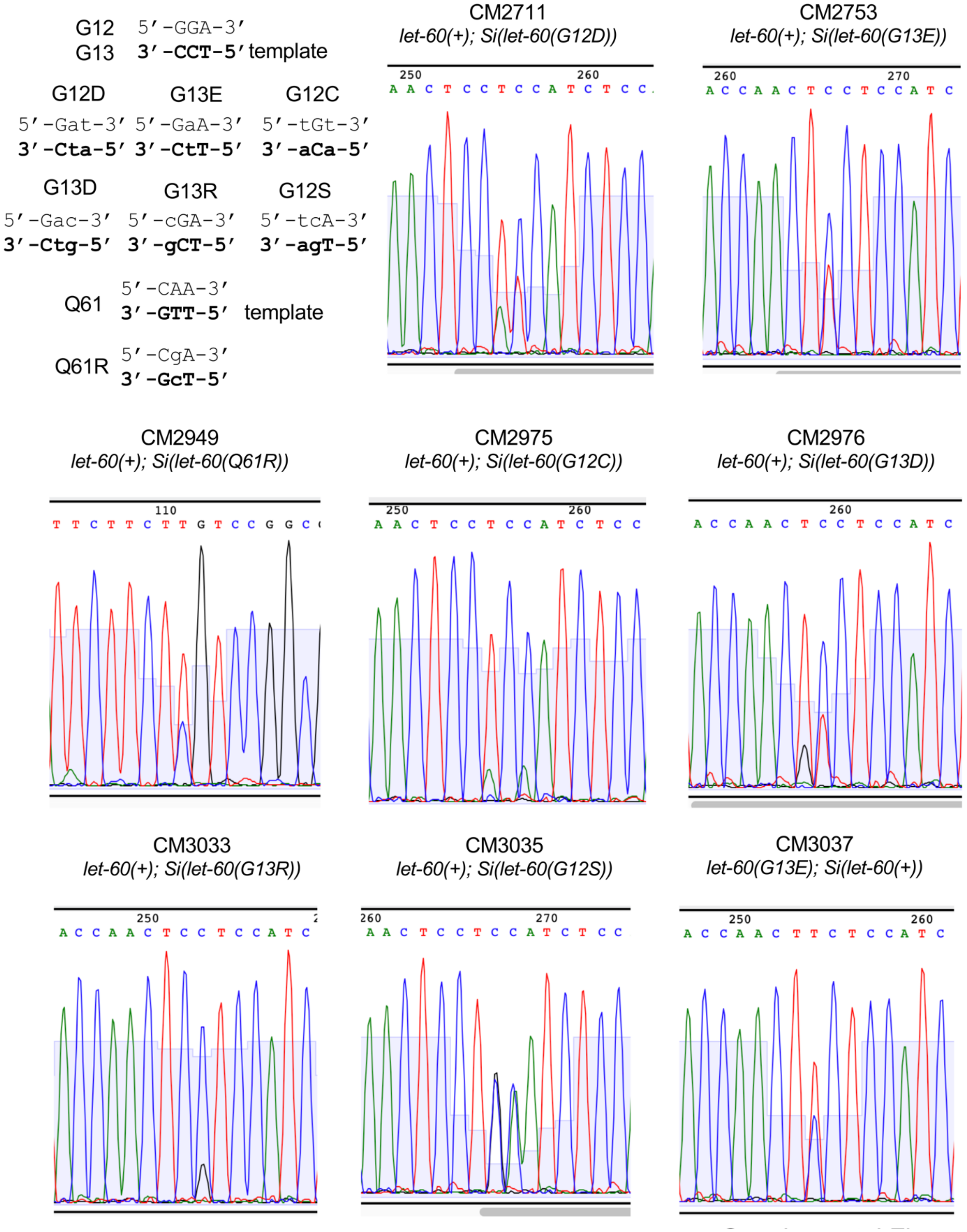

Fig. S3. *guSi(let-60)* variants are expressed *in vivo*. Sanger sequencing traces from *let-60* cDNA recovered from transgene-bearing strains. Sequence corresponds to the codon template strand, as sequencing reads initiated in exon 2 to ensure the products lacked intron 1 (representing cDNA) and high-quality reads were obtained for sequence

Disease Models & Mechanisms • Supplementary information

corresponding to codons 12 and 13. Sequence peaks reflect the relative mixture of transcripts produced by the endogenous locus and the *guSi(let-60)* copies. Consistent with the phenotypic analysis using *let- 60(G13E)* in Fig. 2, the sequence peaks produced from *guSi(let-60)* are less abundant (roughly half) than those from the endogenous locus. In contrast to the others, cDNA corresponding to the *let-60(G12S)* variant in CM3035 is equally abundant as the wild type, suggesting the insertion in this strain is not single-copy.

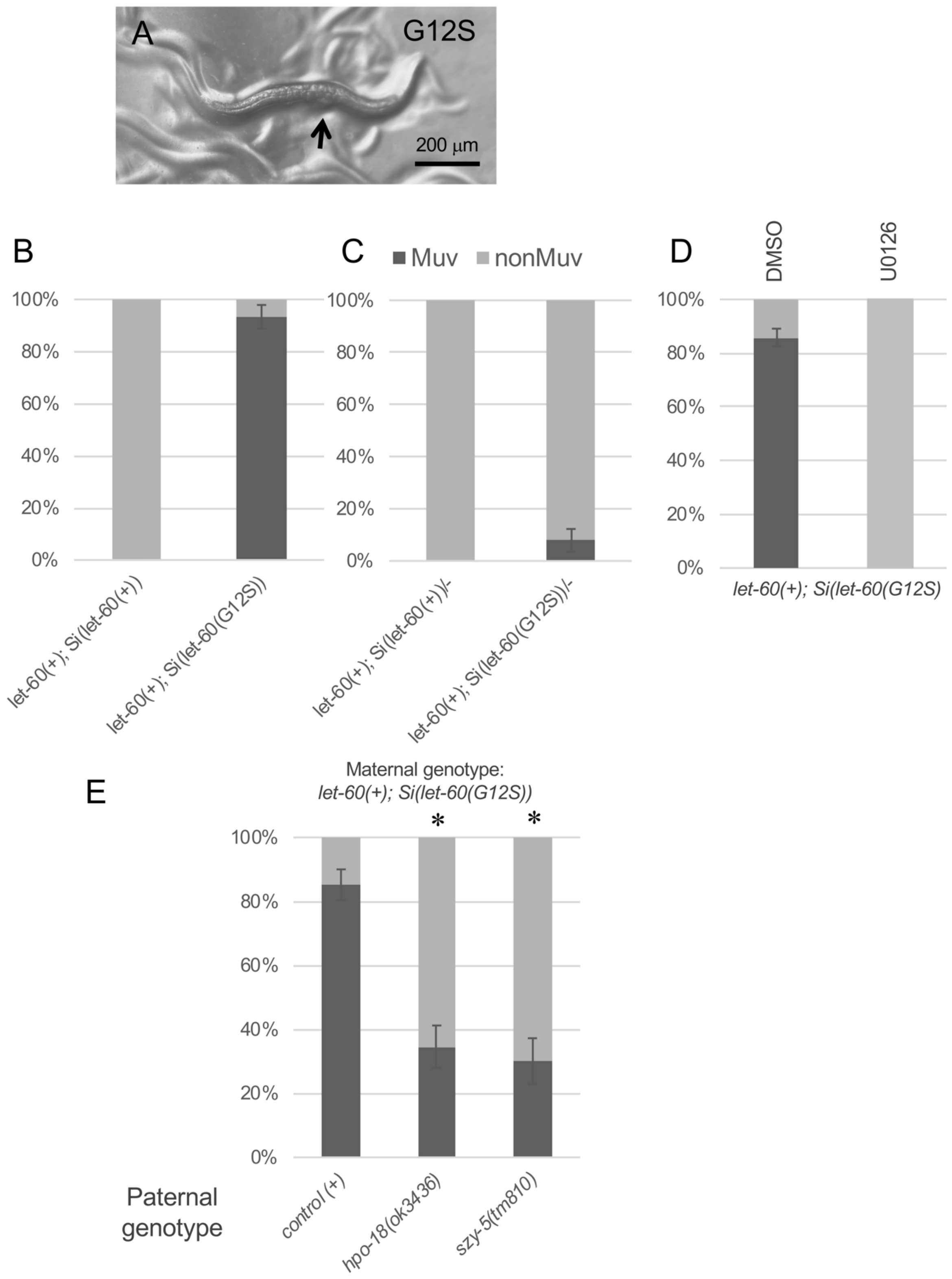

Fig. S4. Phenotypic characterization of a *guSi(let-60(G12S))* transgene. A.,B. The transgene in the CM3035 strain confers a Muv phenotype. Sample sizes in B*: let-60(+); Si(let-60(+))*, 53 (data same as in Figure 3); *let-60(+); Si(let-60(G12S)*, 30. C. The

Disease Models & Mechanisms • Supplementary information

transgene in CM3035 exhibits dose-sensitivity, as hemizygous animals are preferentially nonMuv. Sample sizes: *let-60(+); Si(let-60(+))/-*, 31 (data same as in Figure 4); *let-60(+); Si(let-60(G12S))/-*, 38. D. The Muv phenotype associated with CM3035 is dependent on MEK activity, and animals are sensitive to 60 uM dose of the MEK inhibitor U0126. Sample sizes: 113 (DMSO), 105 (U0126). E. The Muv phenotype associated with CM3035 is sensitive to non-autonomous modulation by *hpo-18* and *szy-5* genotype. Sample sizes: 54 (control (+)), 52 (*hpo-18(ok3436)*), 40 (*szy- 5(tm810)*).

Table S1. Strains used in Lyu and Chamberlin

Available for download at
https://journals.biologists.com/dmm/article-lookup/doi/10.1242/dmm.050577#supplementary-data

Table S2. Primers used in Lyu and Chamberlin

Available for download at
https://journals.biologists.com/dmm/article-lookup/doi/10.1242/dmm.050577#supplementary-data

Table S3. Plasmids used in Lyu and Chamberlin

Available for download at
https://journals.biologists.com/dmm/article-lookup/doi/10.1242/dmm.050577#supplementary-data

Table S4. Sample sizes for data in Fig. 5B in Lyu and Chamberlin

Available for download at
https://journals.biologists.com/dmm/article-lookup/doi/10.1242/dmm.050577#supplementary-data
